# “If you’re struggling, you don’t really care” – what affects the physical health of young people on child and adolescent mental health inpatient units? A qualitative study with service users and staff

**DOI:** 10.1186/s12888-024-05858-1

**Published:** 2024-07-09

**Authors:** Rebekah Carney, Shermin Imran, Heather Law, Parise Carmichael-Murphy, Leah Charlton, Sophie Parker

**Affiliations:** 1 Youth Mental Health Research Unit, Greater Manchester Mental Health NHS Foundation Trust, Manchester, M25 3BL UK; 2 Division of Psychology and Mental Health, Faculty of Biology, Medicine & Health, University of Manchester, Manchester, M13 9PL UK; 3 Greater Manchester Mental Health NHS Foundation Trust, Bury New Road, Prestwich, M13 3BL UK

**Keywords:** Inpatient, Physical health, Qualitative, Adolescent, Child, Mental health

## Abstract

**Background:**

Physical health inequalities of people with serious mental illness (SMI) have been labelled an international scandal; due to the 15–20-year reduction in life expectancy associated with poor physical health. This occurs at an early stage and evidence shows young people with and at risk for SMI are a particularly vulnerable group requiring intervention and support. However, most work has been conducted with adults and little is known about what affects physical health for young people, specifically those receiving inpatient care.

**Methods:**

We conducted semi-structured qualitative interviews with 7 service users and 6 staff members (85% female, age 14–42) on a generic mental health inpatient unit for children and adolescents. Interviews aimed to identify how young people viewed theirphysical health and factors affecting physical health and lifestyle and identify any support needed to improve physical health. Thematic analysis was conducted. .

**Results:**

Thematic analysis revealed the main factors affecting physical health and lifestyle for young people. Three main themes were individual factors (subthemes were mental health symptoms, knowledge, attitudes and beliefs), environmental factors (subthemes were opportunities in a restricted environment and food provision), and the influence of others (subthemes were peers, staff, family members). These factors often overlapped and could promote a healthy lifestyle or combine to increase the risk of poor physical health. Young people discussed their preferences for physical health initiatives and what would help them to live a healthier lifestyle.

**Conclusions:**

Promoting physical health on inpatient units for young people is an important, yet neglected area of mental health research. We have identified a range of complex factors which have an impact on their physical health, and there is a pervasive need to address the barriers that young people experience to living a healthy lifestyle. There is an increasingly strong evidence base suggesting the benefits of physical health interventions to improve outcomes, and future work should identify ways to implement such interventions considering the barriers discussed in this article. Further collaborative research is needed with young people, clinical teams, caregivers, and commissioners to ensure improvements are made to clinical care provision and optimisation of the inpatient environment.

## Background

The poor physical health of people with serious mental illness (SMI) has long been established. People with SMI experience significant physical health inequalities compared with the general population [1–3]. A 15–20-year mortality gap arises from an increased risk of developing non-communicable diseases such as diabetes and obesity, increased likelihood of engaging in behaviours which produce adverse health outcomes, reduced access to and provision of physical health care, and medication side effects [1, 2, 4]. This has been labelled an international ‘human rights scandal’ as much of this risk is preventable [5]. Various national and international health bodies have responded by producing guidelines to reduce the incidence and impact of physical comorbidities in people with SMI. Recommendations include increased access to physical health interventions, implementation of exercise initiatives across clinical settings and improving detection, monitoring, and treatment of physical health [3, 4, 6, 7].

Children and young people with SMI and/or those receiving treatment from mental health services are particularly vulnerable, requiring additional support to look after their physical health and wellbeing. They are more likely to engage in adverse health behaviours such as smoking, less likely to be physically active and consume a balanced diet [8–11]. Individuals on child and adolescent mental health service (CAMHS) inpatient units are particularly at risk for poor physical health, given their restricted living environment, high levels of psychological distress and likelihood of being prescribed antipsychotic medication [12–14]. Our recent meta-analysis of international studies found almost half of young people on CAMHS inpatient units were overweight or obese, and over half smoked tobacco. Concerningly, they also showed early signs of metabolic risk and metabolic syndrome, and high levels of modifiable risk including low levels of physical activity [9]. Although there is increasing evidence to show physical health problems are common in young people with SMI or those receiving CAMHS mental health care, they often go undetected or untreated, and existing guidelines can be unclear or not child focused [10, 15]. Therefore, physical health care is often inconsistent, with staff often lacking clarity over whether it should be their responsibility, meaning more research is needed to optimise physical healthcare.

Despite extensive evidence promoting the use of physical health interventions for adults with SMI [3, 16, 17] there is a paucity of research for young people, particularly in inpatient settings, and little work has been done with a physical health focus in this setting. Our recent systematic review revealed very few physical health interventions had been conducted on CAMHS inpatient units and little is known about the feasibility of implementing such interventions in inpatient settings [14, 18]. However, studies that do exist suggest physical health interventions can improve social functioning, physical health outcomes and quality of life [14, 18–21]. More research is needed to inform policy and practice to improve care provision and identify acceptable ways for implementation in inpatient units. Previous qualitative work with young people with SMI (and those at-risk for SMI) have investigated the barriers and facilitators to living a healthy lifestyle [22–24]. Various psychological barriers such as poor self-efficacy, anxiety and low motivation are often reported, as well as practical issues such as access and financial implications [25]. Young people on inpatient units may also experience these barriers, however, the inpatient environment, despite presenting a unique opportunity to intervene, may pose additional difficulties that need to be considered prior to designing and implementing physical health initiatives.

### Aims

Through qualitative interviews with service users and staff, we aimed to identify:

- How young people view their physical health when on CAMHS inpatient units.
- Factors affecting physical health and lifestyle for young people on CAMHS inpatient units.
- Support that would be useful to help young people on CAMHS inpatient units improve their physical health.

## Methods

This study was reported according to Standards for Reporting Qualitative Studies (SPRQ [26]). Approvals were granted by North-West and Greater Manchester East Ethics Committee (ref:19/NW/0458; August 2019).

### Setting

The study took place within CAMHS inpatient services at Greater Manchester Mental Health NHS Foundation Trust (GMMH NHS FT). The service consists of a 20-bed mixed-gender, adolescent inpatient unit for young people with complex health needs. Individuals are admitted to the unit with severe or acute mental health symptoms meaning they are unable to keep themselves safe. Referrals are received via CAMHS or adult mental health services treating adolescents aged 13–18 years whose needs cannot be met safely within the community, who have a range of diagnoses and mental health needs and experience high levels of psychological distress. Evidence based treatments are provided in line with National Institute for Health and Care Excellence (NICE) guidelines and individuals have access to a range of psychological therapies (individual/group), occupational therapy-based activities and family interventions.

### Participants

Convenience sampling was used. Eligible service users were aged 14–18 and had received inpatient care within the service for at least two weeks. Service users who did not have capacity to consent, had a primary diagnosis of an eating disorder or who had language/communication difficulties were excluded. All potentially eligible participants were given the opportunity to be involved. All staff members were approached who had worked within the service for longer than two weeks. At least two weeks’ experience of the inpatient ward was required to ensure participants had insight into the factors affecting physical health.

### Procedure

Staff were approached at team meetings and through clinical networks with the research team. Staff members were also informed of the inclusion criteria for service users and given information leaflets to give to any potentially eligible participants and obtain consent to contact. Researchers met with any eligible participants to discuss the study and answer any questions. Written informed consent was sought prior to the interview. Service users were reimbursed for their time with a £10 voucher.

### Demographics and sampling

Age, gender, ethnicity, diagnoses, length of stay (service users) and job role (staff) were obtained using a purpose-built demographic form.

Thirteen participants were interviewed (*n* = 7 service users; *n* = 6 staff), See Table 1 for demographics.

**Table 1:** Demographic characteristics of study participants

		Staff (n = 6)	Service Users (n = 7)
Age (mean, range)		29.5 (22–42)	16 (14–16)
Ethnicity	White	6 (100%)	6 (85.69%)
	Black, Asian, Mixed or Other	-	1 (14.29%)
		-	
Gender	Female	4 (67%)	7 (100%)
	Male	2 (33%)	-
Time within the service (mean, range)		25.8 months (12–42 months)	6.3 months (2–24 months)
Diagnoses	Post Traumatic Stress Disorder	-	4 (57%)
	Mood / Adjustment Disorders	-	2 (28.6%)
	Not specified	-	1 (14.3%)
Job Role	Support/Time/Recovery Worker	4 (66.7%)	-
	Allied Health Professionals	2 (33.3%)	-

### Qualitative interviews

A qualitative design was employed using semi-structured interviews. Topic guides were developed by the study team based on previous research and consultations with young people within the service [15, 27] (topic guides available on request). Semi-structured interviews were conducted by the lead author and psychiatrist within the clinical service. They covered a range of pre-specified topics about physical health for young people within the service. This included questions about diet, exercise, and physical health care. They were also asked about barriers and facilitators to living a healthy lifestyle on inpatient mental health units, as well as their beliefs about physical health, and what would help promote physical health in the inpatient environment. The interview schedules were adapted to staff and service users and lasted approximately 1-hour. Interview guides were flexible, used prompts and open-ended questions to encourage participants to talk in-depth about their experiences. Interviews were recorded on an encrypted dictaphone and transcribed verbatim for analysis. Pseudonyms were used to maintain anonymity.

### Qualitative analysis

There were some pre-specified areas of interest which included identifying the main barriers and facilitators to living a healthy lifestyle on inpatient mental health units, and how to optimise physical health care. This means that we aimed to identify the main themes in these areas which came from the data. Thematic analysis was conducted on the transcripts to analyse the data. Thematic analysis is a systematic approach whereby patterns and common themes are identified to describe a data set and understand more about a given phenomenon [28]. An inductive approach was adopted to identify common themes in the data, according to Braun and Clarke’s (2006) method which was conducted as follows:

1. Transcripts were read and re-read by researchers until they were familiar with the data and could anticipate what the respondent would say next.
2. Researchers systematically coded line by line to identify common features in the data.
3. Codes were reviewed to determine potential themes.
4. Themes were reviewed through discussion for internal homogeneity and external heterogeneity and ensure they were distinctive and rational.
5. Themes were defined and named.

All researchers were involved in the analytic process, and transcripts were coded individually by multiple researchers. Data analysis was conducted using nVivo (Version 12, Qualitative Data Analysis Software, 2015). Several processes were followed to ensure trustworthiness. Credibility was achieved by researchers adhering to a set protocol and following the rigorous methods as described by Braun and Clarke above. Themes and codes were discussed throughout, as well as how authors predispositions may be affecting decisions about codes and themes and all discrepancies were resolved through discussion to ensure confirmability. Quotes are presented within the results section to illuminate findings and add context to themes. Codes from the two groups were synthesized to identify overarching themes. To ensure transferability a detailed description of the service and setting has been reported and people with clinical expertise and knowledge of CAMHS were involved in the analytic process to ensure validity.

## Results

### Factors affecting physical health

**Fig. 1:**
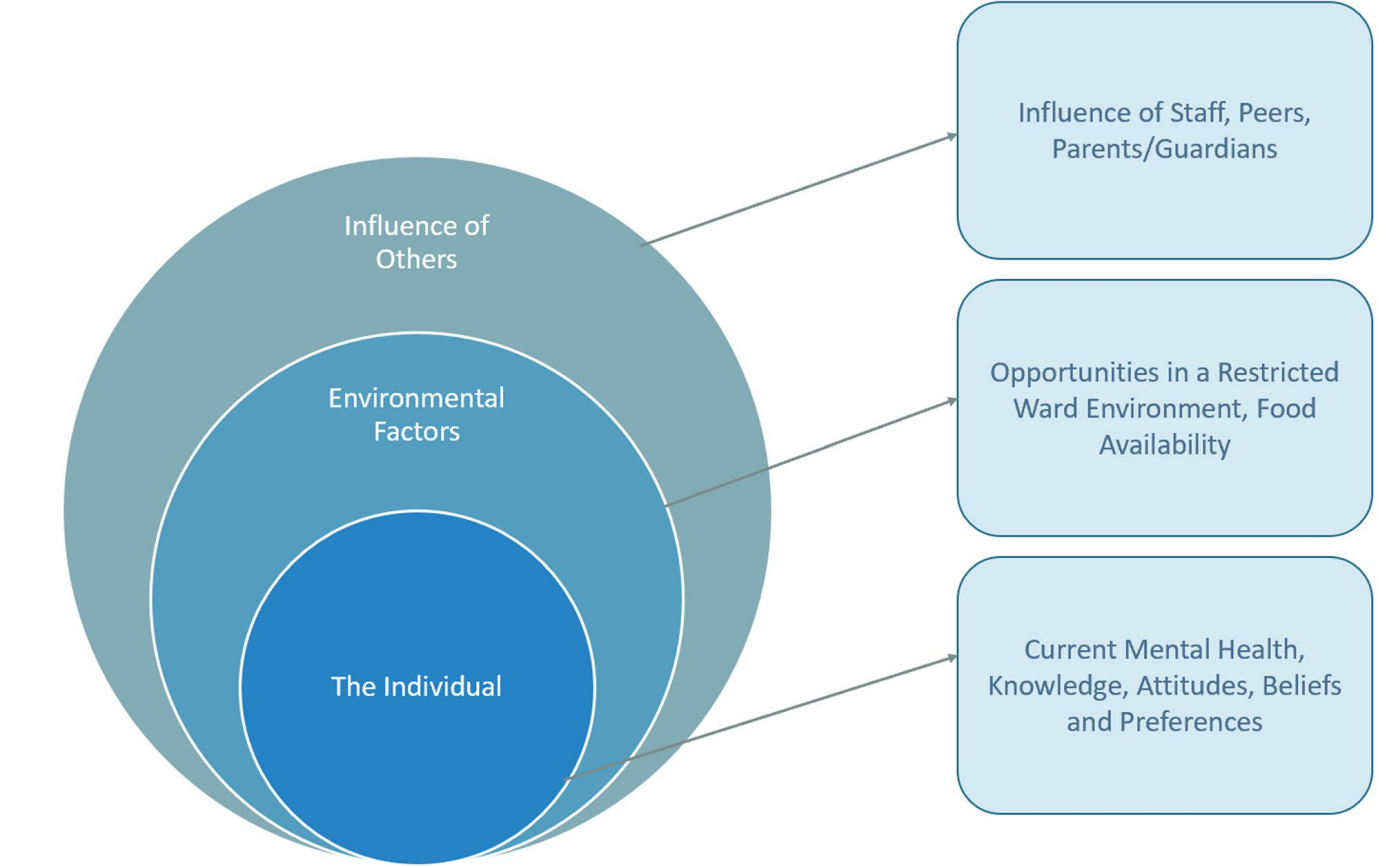
Factors affecting physical health of young people on CAMHS inpatient units

A wide range of factors affecting physical health were discussed. They could be broken down into three themes: “Individual Factors”, “Environment”, and “Influence of Others”. See Table 2 for supporting quotes, and Fig. 1 for a descriptive diagram.

**Table 2:** Quotes of key themes

1. INDIVIDUAL FACTORS		
Current Mental Health Symptoms	YP1 “I think it’s the fact that when people come into hospital they are at a low point, so they won’t have the motivation, they won’t think. They might not be able to keep themselves safe, they might not want to do exercise. They might just think completely no, I don’t want to look after myself”. YP7"I think it’s the medication because the medication makes you really hungry. That’s unhealthy because you would be overeating”. YP4:“sometimes I just you know, just can’t get out of bed and don’t want to do anything”*	ST1: “you get a lot of the young people saying oh I’m putting on so much weight cause it’s like the medication sometimes, or just the fact that they are in here and they’re not doing that much, so they may just be eating and may be putting on weight” ST2:“are just quite socially withdrawn and would just feel more comfortable spending all of their time in their bed space, and they might not feel comfortable in big groups” ST2:“young people with a first episode psychosis who will then go on medication that is very sedative. So, they will spend like all day in bed, or young people with low moods so they will spend all day in bed, that is hard to get them to get up and about”. ST1 “putting on that weight in maybe a short period of time can be really like debilitating for them… you’ve got a lot of them that wear long sleeves and pants, won’t wear shorts or anything like that and that was big sort of barrier with the running group because they wanted to cover up and they were embarrassed about how they looked or how they ran.” ST6:“one of the lads currently he is on medication which increases his hunger, and he is saying like I’m eating 8 loads of bread in between dinner and supper… and he is quite paranoid about putting on weight”*
Knowledge	YP3: “I think it has really positive impact on mental health because like I said before it does increase your mood because I’ve noticed when I been, say to the gym I’ve finished I’ve come out feeling so much better and have a lot more energy” YP6:“…because they always on their phones and I kind of feel like yeah, its ok to be on your phone should but you should actually take some time to exercise”. YP4:“how to cook healthier meals…. what kind of exercises are the best”.* YP3:”run some like training like first aid, like little programmes for young people to do, so that we can like take charge of our own and maybe like might know how to manage our own physical health”*	ST4:“I don’t think they have got the understanding I think they think well, I have eaten a salad so I can have a chocolate bar. That’s the mentality around it, so I think they need more education on what certain foods give for you and how that then impacts on other things.” ST4:“I think it’s the lack of knowledge about food actually, you know what, it’s what sort of it is really you know as opposed to yeah, I just fancy that, or well I didn’t eat anything yesterday, so I can have 6 cheeseburgers today… that unfortunately I do see that mentality in a lot of them.“* ST3:“people come into us with already unhealthy lifestyles, not a great deal of education around food, diet, exercise.”
Attitudes & Beliefs	YP2:“…sometimes I think about it but then like if you’re struggling you don’t really care, so at other times there may be other things you are more worried about and you may not think about physical health”. YP1:“I think it is quite important, but I think even though it is important, it doesn’t really mean you can do anything about it”.	ST3:“In terms of their actual physical health, I don’t think it’s at the top of their priorities. Body image more so, but actually in terms of underlying ‘this is my health, I have to look after my heart’ I don’t think it’s high, in relation… below peer relations”. ST1:” Sometimes they get in their mindset, why bother it’s just going to get cancelled… I think because they are getting their meals cooked for them and brought up, they just don’t see the point”.
2. **ENVIRONMENTAL FACTORS**		
Opportunities in a restricted environment	YP6:“there’s times when people will be ok, but then incidents can bring people down. And there’s times the ward as a whole ward is just purely down, and they don’t want to do anything …. yeah, sometimes actually they can make everyone else upset, and plus the alarms get on people nerves a lot, so people you know have tendency to get agitated, or they get really angry or really annoyed and can’t really calm down”. YP1: " also I think it is like, you’ve got the staff here, so if the staff’s down then you can’t really go out, you can’t go into the gym. I think that there’s loads of factors in the environment, because in the environment things just can’t be helped. Like if there’s an incident and you’re meant to go out then you can’t go out.” YP7:“The walking group it doesn’t happen often as I would like it to and the netball group has stopped as well so there’s nothing much"… “in here generally there isn’t much to do… So what people would do is stay here and eat.“* ST6:“if you haven’t got leave then you’re not even walking anywhere. Your college is at the bottom of the corridor and the dining room is at the door, so it is quite difficult for them. They have got the sports hall, but college do lock quite a lot of it away.“*	ST2 “we’ve also got a sports hall onsite, but it is like just a hall so resources within that aren’t great. so even if people do go there, they are probably not doing very active stuff.” ST2: “this is something that is really quite poor I guess, because the unit itself is not particularly big, so even just going about day-to-day activities not really walking, there’s no stairs so for some young people they might not have climbed stairs in months”. ST4:“If they don’t like the food, they are ordering takeaways… those that don’t have leave, they’ve actually got access to a fair amount of money and …they are not spending it because they are here”. ST3"it was quite hard maintaining the motivation to try and get people doing it regularly”.*
Food Availability	YP1: “yeah I don’t know half of us don’t even like the look of it, so we won’t eat it, and it’s like how can that even be healthy, like you look at it and think how can that be healthy?! Like it doesn’t look very appetising.” YP5: “it does put everyone off… all of us, we hardly eat on this ward… feel like the people who make food for the hospital should put more effort in rather than buying some shop and stuff and reheating it so because if they put effort in then we will try to put effort in eating” YP1:“it’s really hard, doing it here you can’t just go to the gym, … like at home you have like loads of different foods in and you can make your own food here it get brought up"*	ST2:“I guess they are just looking for something that is quick and convenient. “ ST1: “young people they just like change, they don’t like doing the same things every week… the kids are just bored and when the food comes up, like not to be harsh but sometimes it doesn’t look the best and it’s just like, I’m not hungry, I’m not eating it” ST5 “they will go through a phase of like ordering takeaways, and then the ward manager will be like no we need to like pull it back in to one time of a weekend so it will be like a Friday or Saturday night. So, they will order…and pay all together so then the staff member will collect it”. ST2:“nearby things are like Tesco and they will go and buy like chocolates, crisps whatever just cause it’s convenient.”*
3. **INFLUENCE OF OTHERS**		
Staff	YP5:“she come to see you and tell you about food how its healthy and then staff also encourage you to eat even a small bit that will encourage you” YP3:“I don’t always have the motivation to say go out for a run, but the staff do always push me because I always feel a lot better afterwards” YP6: “They don’t like make any suggestions, it kind of like you have to ask them if you want to go to the sport hall or want to do specific physical activities… Yeah you have to ask them it’s not a matter of the staff suggesting it”	ST6:“If staff were a bit more active and a bit more pumped then I think the kids will be…we have proved with the netball when the staff get involved and bring their gym kit the kids get involved”. ST3: “Some people can get on board and other people can be quite resistive at that and feel we are trying to preach or tell them stuff when that’s not our place to do so.” ST1:“We are good at noticing and saying come on let’s go off the ward, let’s go and do this for a little bit and it just totally calms them down, agitation, better mood …we are really good at doing that”. ST5:“one of the new support workers is a yoga teacher and they really like getting involved in that"* ST2:“I guess it’s just difficult because we are not parents, we can’t sort of shout at them and be like that’s bad for you cause they are not going to… that’s like as much as we can do!“* ST4:“Yeah it is difficult, at one point we did try to monitor it and we did initially say takeaways were limited to once a week or on a Saturday you know. But it was very, very, difficult to maintain that because of restrictive practice and you know they are adults, and the capacity, lots of the loopholes prevented us really from actually being able to stop that.”* ST4:“if they’ve got the swimming group… goes down really well here, they do enjoy it. Some of them are reluctant due to body image, which can cause a problem sometimes, in which case we just support with buying more suitable swimwear.“*
Peers	YP3: “getting young people to work together, in say like an exercise group, so like you could make an exercise plan, and then get young people to reflect on it and track it over 10 weeks to see how their mood increased”. YP2:” like some people may think it’s like weird to take part in stuff like that” YP3: “I have quite low self-esteem so sometimes that stops me from wanting to go and play netball, because I’m little bit scared of being judged, and sometimes my mood as well” YP2 " maybe other people might not want to do it so that they can spend time with other people…. they might not want to go out to do exercise because they want to stay on the ward to be friendly and spend time there” YP3:“I think I have quite low self-esteem so sometimes that stops me from wanting to go and play netball, because I’m little bit scared of being judged… I think being encouraged would help, maybe by other young people as well, bit of reassurance”* YP2:“maybe other people might not want to do it so that they can spend time with other people…. they might not want to go out to do exercise because they want to stay on the ward to be friendly”.*	ST1:“I commonly see it could be the dynamic of the group. Say if you go in the running group, I know a lot of the girls who used to come wouldn’t go when a said male was coming"…. could be either due to embarrassment or a bit of just ward dynamics and if they weren’t getting on and didn’t like each other… I find you get one person not like the ringleader, but one person has a lot of influence on the ward so if they won’t take part a lot of the other young people won’t take part”. ST2:“On a ward full of teenagers there’s sometimes quite difficult dynamics, sometimes people will be like, if they are not coming then I am not going, and they won’t join in based on whoever is going.” ST5:“energy drinks as well, that’s a big issue a lot of them will go and get energy drinks because they feel like they need them and a few like think they are a bit cool if they drink them, even though it’s not cause they are really bad” ST6:“I don’t know when you’re a teenager and stuff it’s important what your mates are doing.“* ST3:“I think we do find especially with the activities; you need a strong influence to participate, you know with that one young person who will get on board and then 4 or 5 of the others might come too”*
Parents/Guardians	YP2: “sometimes it’s like healthy sometimes it’s not so like if I am going out with my friends we probably go to the shop and eat a lot of rubbish. But then at home my mum makes me all my food, so she cooks pretty much healthy meals…. like me and my friends get pizza and like McDonalds, and then my mum makes like vegetables and just like you know home cooked stuff “	ST3:“you have people, families who will constantly be bringing in treats and snacks and whether it’s that kind of over caregiving a child who is in hospital, but they’ll bring fast food, KFC, Macy D’s, or just sweets and reems of snacks… “get people who possibly bring in more than you usually would at home, or possibly that’s their response to trying to elicit that care and affection for their child and that’s what…"” ST6:“parents come they tend to bring really unhealthy things because they think it is sort of like he’s in hospital, here’s some nice things. Like we can’t control that but when you do say can you please bring something healthier in they still bring lots of multipacks of crisp, pop, sweets. And the kids over here just can’t be trusted. Like will eat a big chocolate bar.”

*Example quotes included in the manuscript

## Individual factors

The first theme was ‘Individual Factors’ which was defined as anything that was associated with the young person as an individual and linked to their personal experiences. This was broken down into specific subthemes such as their current mental health, knowledge, attitudes and beliefs.

### Current mental health symptoms

Current mental health symptoms had a major impact on physical health and lifestyle. Symptoms of depression such as low motivation, apathy/disinterest, and a preference to stay in their rooms affected physical activity levels.

YP4:“sometimes I just you know, just can’t get out of bed and don’t want to do anything.

They described feeling anxious, particularly in a social group, and preferring to be alone. Social withdrawal was common and meant they sometimes avoided group activities such as running/walking/smoothie making.

Staff discussed direct links between mental health and physical health, via side effects of medication. The theme of weight gain was prominent, with staff attributing this to metabolic side effects such as *“increased appetite”* and *“sedative effects”* reducing energy and motivation. Young people also reported gaining weight since being on the unit, that medication *“makes you really hungry”*, and that they were more self-conscious, and unhappy with their appearance.

ST6:“one of the lads currently he is on medication which increases his hunger, and he is saying like I’m eating 8 loads of bread in between dinner and supper… and he is quite paranoid about putting on weight.

### Knowledge, attitudes and beliefs

Young people were aware of some of the benefits of living a healthy lifestyle (exercising and eating a balanced diet). They reflected on personal positive impacts such as improved mood, *“feeling so much better and having a lot more energy”YP3*. Yet, achieving this was difficult, and they described many barriers to doing so including feeling unable to live a healthy lifestyle. When asked about their understanding, many referred to exercising, eating a balanced diet, getting enough sleep, and avoiding smoking and alcohol use. However, descriptions were often vague, and some struggled to provide examples. This was reinforced by staff who claimed that young people had basic knowledge and skills (e.g., ability to cook a meal), but less awareness of the importance of a healthy lifestyle.

ST4:“I think it’s the lack of knowledge about food actually, you know what, it’s what sort of it is really you know as opposed to yeah, I just fancy that, or well I didn’t eat anything yesterday, so I can have 6 cheeseburgers today… that unfortunately I do see that mentality in a lot of them.”

Young people expressed their preferences for a dynamic approach to exercise, with a variety of options (e.g., groups such as yoga, netball, football). Making sure activities were accessible for everyone was also important, via an individualized approach based on “*what people love and enjoy”* and activities that *“make it look fun and easy”* so anyone can take part. There was also a desire from both young people and staff to pursue activities that improve their knowledge and skills, and promote autonomy over their physical health.

YP4:“how to cook healthier meals…. what kind of exercises are the best”.

YP3:“run some like training like first aid, like little programmes for young people to do, so that we can like take charge of our own and maybe like might know how to manage our own physical health”.

Physical health was viewed as less important than their mental health. Although many acknowledged they could be doing more, physical health was not described as being *“at the top of their priorities”* and young people claiming “*if you’re struggling you don’t really care*”. Similarly, other activities held higher value, such as engaging in social activities or leaving the ward (including “*the cinema*” or “*bowling*”).

## Environmental factors

The second theme was ‘Environmental Factors’ which was defined as anything that affected young people as a direct result of living and receiving treatment on a CAMHS inpatient unit and their wider environment. Specific subthemes related to environmental restrictions of the unit, included opportunities available to engage in activities in a restricted environment defined as lack of access to outdoor spaces the food provision and the changing dynamics of the environment and pressures on staff.

### Opportunities in a restricted environment

A range of facilities were available including a sports hall, exercise equipment, and a communal kitchen. However, access was limited, for example, “*sport stuff was locked because it was reserved for college”*. Some individuals could not leave the unit and would be reliant on ward-based activities, which required staff availability. However, staff claimed *“the main problem is trying to get people engaged”* particularly for those who did not have leave. Therefore, young people stressed the importance of making sure there is an *“activity for everyone”* on and off the wards. However, staff claimed they would get bored easily, particularly if they did not have anything to aim towards, or were given repetitive tasks.

ST3"it was quite hard maintaining the motivation to try and get people doing it regularly”.

A discrepancy occurred between the views of staff and young people. Staff claimed activities were *“always off the cuff, informal and really does benefit”* because “*they’ve got options, but they get bored of them really quick”*, compared with structured activities where attendance at groups was not guaranteed. However, this contrasted somewhat with the views of young people who although did have a desire for a “*variety”* of activities, they wanted consistent and organized activities. However, activities were viewed as inconsistent and sometimes cancelled at short notice meaning young people thought “*why bother it’s going to get cancelled”*, thus reducing their willingness to engage.

YP7:“The walking group it doesn’t happen often as I would like it to and the netball group has stopped as well so there’s nothing much"… “in here generally there isn’t much to do… So what people would do is stay here and eat.”

Physical restrictions and limited space in a smaller, confined environment resulted in individuals being more inactive, and therefore, day-to-day activity was limited.

ST6:“if you haven’t got leave then you’re not even walking anywhere. Your college is at the bottom of the corridor and the dining room is at the door, so it is quite difficult for them. They have got the sports hall, but college do lock quite a lot of it away.”

The ward environment was also described as unpredictable, with frequent admissions and discharges, and a changing presence of young people/staff. Planning activity sessions was difficult, despite attempting to fit within the usual ward schedule, they were often inconsistent, and attendance varied. Young people claimed incidents occurred frequently, which had a detrimental effect on their ability to engage in activities for example, “*if there’s an incident and you’re meant to go out then you can’t go out”.*

### Food availability

Food came from the hospital catering department, as well as items bought on leave or brought in by visitors. A negative view was taken of the food available with frequent descriptions of food being:

beige”, “bland”, “stodgy”, “carby”, “unhealthy”, “pre-packaged crap”, “reheated.

Healthier options were provided, yet were described as unappealing (e.g., “*limp salads*”). Young people claimed “*the choice of food would stop someone eating a healthy diet”* and that “*half of us don’t like the look of it so won’t eat it”.* They wanted fresh and healthy home-cooked food, and the opportunity to prepare meals themselves. Staff claimed that “*There is the option for us to let young people choose their meals, it’s just very difficult when the ward is so busy”* and therefore it was not always feasible to achieve this as young people may be unavailable when the order needed to be made or staff were under clinical pressure. Staff liaised with catering to make specific requests; however, time did not always permit this approach, and many described ordering “*quick*” and “*safe options*”, acknowledging that it was not the healthiest, but what they knew would get eaten, e.g., pizza, chips, burgers, pastas. Snack foods such as biscuits/toast were also readily available, and although fruit was provided staff claimed young people avoided it.

Although kitchen facilities were available, staff claimed it was not always easy to use due to time/funding restrictions, and practical barriers such as “*missing equipment*”. Staff also claimed it required “*a lot of hoops to jump through*”. This is despite them reporting high levels of enjoyment and engagement in cooking activities, and young people wanting “*fresh home-cooked food*” options. Additionally, for those who were able to leave the opportunities to purchase food were limited due to the hospital location being “*surrounded by fast-food places”* and a local supermarket.

ST2:“nearby things are like Tesco and they will go and buy like chocolates, crisps whatever just cause it’s convenient.

YP1:“it’s really hard, doing it here you can’t just go to the gym, … like at home you have like loads of different foods in and you can make your own food here it get brought up”.

## Influence of others

The third theme was the influence other people had on young people’s physical health and how this could be both positive and negative and included subthemes of staff members, peers, and family. Receiving encouragement, guidance and advice was a supporting factor for physical health. For example, being guided to make better food choices, encouraging physical activities or helping them attend group exercise sessions by alleviating worries or concerns.

“*YP3:“being encouraged would help, maybe by other young people as well, bit of reassurance”*”.

### Staff

Staff attitudes had a significant effect. When staff displayed an interest and passion for physical health, exercise, or nutrition, this had a positive impact on young people and the ward environment.

ST5:“one of the new support workers is a yoga teacher and they really like getting involved in that”.

Working collaboratively, such as staff and service users exercising together was seen to *“break down barriers”*, as they would “*all look like each other”*. Examples included netball tournaments for staff and young people, group cooking or smoothie sessions, and walking/running groups.

Staff beliefs about their job role had an impact, some claiming physical health was not their responsibility or their role. They also described feeling a lack of control over young people and their actions; a dynamic which was further compounded by young people being treated as adults, with staff wanting to avoid too many restrictions, but equally being limited by trying to adopt a caregiver role in the absence of a parental figure.

ST2:“I guess it’s just difficult because we are not parents, we can’t sort of shout at them and be like that’s bad for you cause they are not going to… that’s like as much as we can do!”

One example discussed by many of the staff related to the ordering of takeaways, and the lack of control staff felt like they had, some referring to young people as ‘adults’, despite being under 18.

ST4:“Yeah it is difficult, at one point we did try to monitor it and we did initially say takeaways were limited to once a week or on a Saturday you know. But it was very, very, difficult to maintain that because of restrictive practice and you know they are adults, and the capacity, lots of the loopholes prevented us really from actually being able to stop that.

When staff were available and could provide support and encouragement this was seen as particularly valuable, for example *“the staff push me, and I always feel a lot better afterwards*”. Staff claimed they tried to encourage young people by being responsive to their needs and problem-solving barriers.

ST4:“if they’ve got the swimming group… goes down really well here, they do enjoy it. Some of them are reluctant due to body image, which can cause a problem sometimes, in which case we just support with buying more suitable swimwear.”.

However, ongoing clinical pressures meant they were not always able to do their job to the best of their ability and got pulled into different duties. This meant that activities such as exercise groups were cancelled, suggesting that physical activities were not a clinical priority.

### Peers

Peers had a profound impact on the behaviors of young people. They valued encouragement and validation from others, wanting to fit in, to not be seen as “*weird*” or “*outcast”* if they behaved differently to their friends and worrying about being judged. They were conscious of how they were perceived by others and being accepted by their peers through *‘doing what everyone else is doing’*. This was echoed by staff who claimed they were doing what *‘normal teenagers do’*, particularly in the context of following trends and being influenced by peers.

ST6:“I don’t know when you’re a teenager and stuff it’s important what your mates are doing.“

YP3:“I think I have quite low self-esteem so sometimes that stops me from wanting to go and play netball, because I’m little bit scared of being judged… I think being encouraged would help, maybe by other young people as well, bit of reassurance.

Group dynamics significantly affected participation in groups. Staff described “*difficult dynamics”* and “*if one person… has a lot of influence on the ward, if they won’t take part a lot of the other young people won’t take part”.* Similarly, if someone stopped attending a group it would have a detrimental impact on engagement, highlighting how impressionable peers were.

ST3:“I think we do find especially with the activities; you need a strong influence to participate, you know with that one young person who will get on board and then 4 or 5 of the others might come too.

The risk of not being accepted in a peer group played a role in what young people saw as important activities, sometimes preferring to stay in communal areas to socialize, rather than engaging in health activities.

YP2:“maybe other people might not want to do it so that they can spend time with other people…. they might not want to go out to do exercise because they want to stay on the ward to be friendly”.

### Parents/Guardians

The influence of parents/guardians was discussed, particularly in relation to bringing in food. Staff stated parents often brought in *“really unhealthy food”* or treats in an act of *“over caregiving a child who is in hospital”* to “*elicit care and affection”.* Examples of these included *“share-size chocolate bars”, “fast-food”, “multipacks of crisps”, “sweets*”. Some young people described at home their parents would provide them with healthier home-cooked foods, compared to when they are eating with their peers, for example, “…*me and my friends get pizza and like McDonalds, and then my mum makes like vegetables and just like you know home-cooked stuff”*. They suggested the food was different at home when cooked by their parents to what they have access to in hospital, or when with peers which was not as fresh/home cooked. However, it could also be an improvement for people who had a difficult home environment, as it meant they had access to regular hot food.

## Discussion

The aim of this study was to conduct a qualitative exploration of the physical health of young people on CAMHS inpatient units, specifically identifying what factors affect it and what support would be helpful. Young people on mental health inpatient units experience multiple complex factors which contribute to the onset of poor physical health. The main themes were individual factors (such as their attitudes, knowledge, beliefs, mental health), influence of others (peers, staff, family), and the complexities of the inpatient environment. Young people and staff suggested ways to optimise the inpatient environment and identified approaches which may benefit their physical health.

**Fig. 2:**
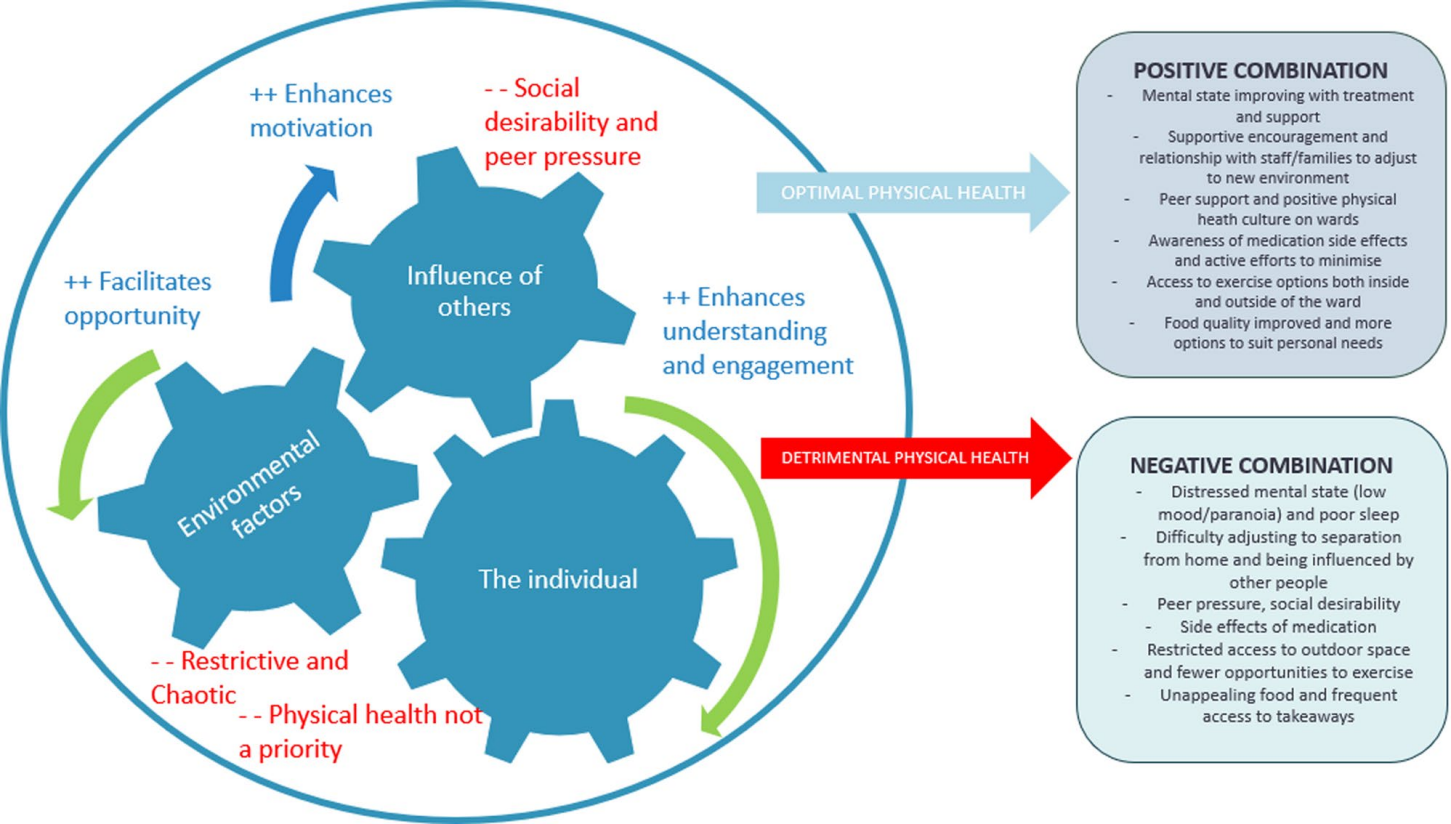
Combination of factors and impact on physical health

The factors influencing physical health were complex with young people central to all internal and external influences (see Fig. 1 for an overview of theme names and subthemes). This fits within an ecological systems theory, which explains how an individual’s development and behaviour is shaped, given the influence of other people, the environment, policy, and societal systems [29]. This can map on to our findings and themes of individual factors (such as mental health, attitudes and beliefs and knowledge), the CAMHS inpatient environment and the influence of other people namely peers, care teams and families. Considering how these factors combine can help understand and explain the main underlying issue, which is the reason why young people on CAMHS inpatient units are at increased risk for physical co-morbidities. For example, if the environment is restrictive, chaotic, and inconsistent, people exert negative influence through peer pressure/lack of encouragement and if the young person has low motivation, poor mental health will only exacerbate the risk of developing physical health conditions. Conversely, looking at how these factors interact in a positive and conducive way can result in important recommendations to optimise clinical practice and care for young people (See Fig. 2 for an example of how these factors may combine to result in a positive or negative outcome).

### Clinical implications

This study has important clinical implications. It adds to ongoing research which suggests that young people on inpatient units face a myriad of challenges to living a healthy lifestyle [9, 15, 18, 30]. Given the high rates of premature mortality and preventable ill-health experienced by people with SMI, it is imperative that we address this inequality for young people. Our study adds further concern regarding the ‘obesogenic’ nature of inpatient environments [12, 13]. Yet, it is important to note many barriers are modifiable, and can be improved through changes to policy, practice, and optimisation of health care provision. Admission to an inpatient unit is a distressing time for individuals, yet it is also a key opportunity to intervene to change lifetime behaviours. The structured nature of the environment can be used to an advantage whereby care providers have the control to equip young people with life skills and knowledge. According to the WHO, physical health behaviours adopted during adolescence (such as poor diet, smoking, inactivity), are likely to persist into adulthood [31]. For example, during adolescence sedentary behaviour often replaces light activity, and increases the risk of depression later in life [32].Therefore, it is essential that physical health is considered in inpatient units to avoid iatrogenic harm, for example, by increasing physical activity levels.

There are many factors relating to staff which carry important clinical implications. There were several instances where the views of young people contrasted to staff, (such as structured approach compared to ad-hoc), and where staff views conflicted, claiming they wanted to treat young people as adults but feeling like they had no control over their behaviours. This can be explained using the principles of attachment theory which argues the importance of secure attachments during adolescence particularly in a post pandemic world [33], as well as previous research of mental health recovery in young people [34]. Additionally, in line with research in other clinical populations [22, 35–37], some staff did not view physical health as part of their job role/responsibility in a mental health setting. This suggests a further training/education is needed for mental health professionals targeting confidence and attitudes towards physical health. An example of where this has been successful is the ‘Keeping our Staff in Mind’ study where a brief lifestyle intervention for staff had a positive impact on physical health outcomes in both staff and young people [38, 39]. Additionally, staff have a duty of care as the main caregiver in the absence of a parental figure to support individuals with mental and physical health. Our findings suggest that young people value the encouragement and support from staff, despite wanting to become autonomous. Future work should build on this to improve the ward culture through equipping staff with skills and modelling positive behaviours for young people through shared dining and getting involved in physical activities, whilst also engaging in collaborative planning through discussions on the ward.

### Clinical recommendations

Table 3 contains several examples of clinical recommendations to optimise service provision and care for young people, using some specific examples from the themes and subthemes which we found in our study.

**Table 3:** Clinical and research recommendations

“I think it’s good, but I think there’s room for improvement so that you can give better care.” YP2				
**Example of Barrier**	**Related Theme (s)**	**Quote**	**Clinical Recommendation**	**Research Recommendation**
Conflicting views of YP and staff around delivery of physical health initiatives e.g., vs. structured approach.	Environment (Opportunities in a restricted environment)	ST1 “I’ve worked in adults before and I think structure with adults works really well, but I think definitely working at junction 17 you’ve got to be off the cuff”. YP7 “The walking group it doesn’t happen often as I would like it to and the netball group has stopped as well so there’s nothing much"… “in here generally there isn’t much to do, do we’re not sure… so what we can do is eat. So, what people would do is stay here and eat.”	Collaborative planning of activities with staff and young people to discuss what groups would be needed and including them on care plans and on the ward planner. To identify the need for staff training to deliver these activities safely and provide this training.	Audit of activities that had been planned and delivered, and undertake mapping of reasons why they did not take place. Focus groups to be held with staff to explore the barriers. Identify successful pilot schemes/programmes and work with patient and public involvement to identify best ways to implement interventions.
Parents/guardians providing access to takeaways and unhealthy food.	Influence of Others (Parents/Guardians)	ST1 “. a lot of the times we do get parents bringing in…KFC because we are surrounded by fast food places round here. They will bring in McDonalds and we have to really try and watch that and try and discourage that because they will just bring in piles and piles of like junk food and takeaways. And that’s difficult because we can’t search every visitor, we can’t keep count on what they are bringing in.”	Placing restrictions on foods that are allowed to be brought on to the unit and including information in letters to parents/guardians about the importance of good nutrition.	Creating an educational intervention on promoting physical health for young people when on inpatient units, e.g., explaining the link between food and mental health, the side effects of medication.
Staff feeling a lack of control over young people’s behaviours such as ordering takeaways.	Environment (Food Availability)	ST5 “…they’ve got the freedom to do that, they go on their phone, and they’ll order … and then be like can you go and get my takeaway from the door, and then that’s … we don’t really have much control over it. Sometimes they’ll ask and we will be like no it’s not a weekend because generally we will say on a Friday, they can order a takeaway but then sometimes they will just be like I’ve ordered takeaway.”	Development of guidelines around takeaway provisions. Staff could work with young people to cook ‘fakeaways’ on the ward kitchens to provide alternatives to ordering takeaways.	Further qualitative work with staff to explore the conflict of beliefs around controlling the behaviour of young people, as well as the development of training programmes which consider the principles of adolescent development. Identify with staff what the perceived barriers are to cooking activities on the ward to prevent ordering takeaways, such as provision of cooking equipment and training.
Peer influence and wanting to fit in with others.	Influence of Others (Peers)	ST1 “And sometimes you get I think I find you get one person not like the ringleader but one person has a lot of influence on the ward so if they won’t take part a lot of the other young people won’t take part”. YP3 “I think I have quite low self-esteem so sometimes that stops me from wanting to go and play netball, because I’m little bit scared of being judged… I think being encouraged would help, maybe by other young people as well, bit of reassurance”.	Identifying peers who have a strong influence on the ward to engage them in health activities and appoint volunteers as physical health ‘champions’ to promote engagement with activities.	Assessing the impact of peer support on inpatient units to promote physical health behaviours.
Staff attitudes and Beliefs towards mental health and a confusion around whose responsibility it is to provide physical healthcare.	Influence of Others (Staff)	ST6 “increasing staff motivation on the wards and push for the activities to engage with them. Staff aren’t the only thing, but it is really important if staff get involved then the kids will get involved so, improving sort of staff culture around doing the sports themselves as well”	Providing further staff training in adolescent development. Implementing clear job roles and responsibilities for monitoring physical health.	Implementing a staff training programme such as the KOSIM in physical health which seeks to promote a more positive physical health environment.

### Strengths and limitations

To the best of our knowledge this is the first study to consider opinions of both young people and staff on CAMHS inpatient units. Qualitative analysis gives a deep and rich account of the data. This provides us with important clinical information and has real world implications, adding to the growing movement to improve physical health of people with SMI. However, our study sample was not representative of the area the trust serves, or the range of occupations at the trust. For example, we did not manage to recruit any male service users, however, at the time service users were mainly female. Additionally, interviews were conducted in one hospital, which means operational barriers may differ nationally and internationally. Some of the interviews were conducted by a CAMHS consultant psychiatrist which has the potential to affect responses and introduce a power imbalance. However, the psychiatrist involved in the study was not directly involved in clinical care and decision making for these service users. Additionally, in an attempt to mitigate any other risk of bias, all participants were notified in advance and informed that they could request to be interviewed by another member of the research team. Furthermore, this study took place prior to the covid pandemic, which has resulted in changes to clinical care delivery [40, 41]. However, the growing evidence base suggests our findings may have utility to other health providers.

### Future research

There is an imperative need to address the barriers that young people experience to living a healthy lifestyle on inpatient units. There is an increasingly strong evidence base arising to show the benefits of using physical health interventions to improve outcomes for young people [9, 14, 18]. Future work should explore the development and implementation of initiatives considering the views of young people, clinicians, caregivers, and commissioners. This may include changes to clinical practice and procedures to remove operational barriers, and development of training/education programmes. Further attention should be given to address the conflicting views of young people and staff, by sharing research findings and encourage collaborative working. Any future work should adopt a developmental approach, due to the overarching influence of adolescence discussed in the interviews (such as the high value placed on peer influence and social desirability). This is a well-known critical factor included in public health programmes for young people for example, in areas such as sexual health, substance use, drink/drug driving awareness, knife crime and anti-bullying [42]. Therefore, there is a need to identify how to balance factors related to adolescent development such as increasing autonomy, independence, and choice, whilst allowing and encouraging staff to guide and shape the choices young people are making, acting as therapeutic caregivers in the absence of parents/guardians.

## Conclusions

There is an urgent need to develop interventions to reduce the risk of young people developing preventable illness and disease and improve long-term physical health outcomes. Young people’s physical health is affected by multiple factors, and fall into three main themes individual factors, the environment and influence of others. Clinical teams cannot care for mental health without considering physical health as the two are intrinsically intertwined, therefore it is vital that the barriers identified in this study are addressed, and suggestions for clinical improvements are explored. More work is needed, including collaborative research with young people and clinical teams, improvements to clinical care provision and optimisation of the inpatient environment. This will ensure all young people with SMI will have the opportunity to live an active, healthy, and fulfilled life, and ultimately reduce the inequality gap in physical health care provision.

## Data Availability

The datasets analysed during the current study are available from the corresponding author should a reasonable request be made.

## Abbreviations

CAMHS: Child and Adolescent Mental Health Services
CYP: Children and Young People
GMMH NHS FT: Greater Manchester Mental Health NHS Foundation Trust
NHS: National Health Service
NICE: National Institute for Health and Care Excellence; SMI: Serious Mental Illness
WHO: World Health Organisation
